# Coordination between Prefrontal Cortex Clock Gene Expression and Corticosterone Contributes to Enhanced Conditioned Fear Extinction Recall

**DOI:** 10.1523/ENEURO.0455-18.2018

**Published:** 2018-12-21

**Authors:** Elizabeth R. Woodruff, Lauren E. Chun, Laura R. Hinds, Nicholas M. Varra, Daniel Tirado, Sarah J. Morton, Colleen A. McClung, Robert L. Spencer

**Affiliations:** 1 Department of Psychology and Neuroscience, University of Colorado Boulder, Boulder, CO 80309; 2 Department of Psychiatry, University of Pittsburgh Medical Center, Pittsburgh, PA 15219

**Keywords:** circadian, clock gene, conditioned fear, corticosterone, prefrontal cortex, PTSD

## Abstract

Post-traumatic stress disorder (PTSD) is associated with impaired conditioned fear extinction learning, a ventromedial prefrontal cortex (vmPFC)-dependent process. PTSD is also associated with dysregulation of vmPFC, circadian, and glucocorticoid hormone function. Rats have rhythmic clock gene expression in the vmPFC that requires appropriate diurnal circulatory patterns of corticosterone (CORT), suggesting the presence of CORT-entrained intrinsic circadian clock function within the PFC. We examined the role of vmPFC clock gene expression and its interaction with CORT profiles in regulation of auditory conditioned fear extinction learning. Extinction learning and recall were examined in male rats trained and tested either in the night (active phase) or in the day (inactive phase). Using a viral vector strategy, *Per1* and *Per2* clock gene expression were selectively knocked down within the vmPFC. Circulating CORT profiles were manipulated via adrenalectomy (ADX) ± diurnal and acute CORT replacement. Rats trained and tested during the night exhibited superior conditioned fear extinction recall that was absent in rats that had knock-down of vmPFC clock gene expression. Similarly, the superior nighttime extinction recall was absent in ADX rats, but restored in ADX rats given a combination of a diurnal pattern of CORT and acute elevation of CORT during the postextinction training consolidation period. Thus, conditioned fear extinction learning is regulated in a diurnal fashion that requires normal vmPFC clock gene expression and a combination of circadian and training-associated CORT. Strategic manipulation of these factors may enhance the therapeutic outcome of conditioned fear extinction related treatments in the clinical setting.

## Significance Statement

Posttraumatic stress disorder is associated with deficits in conditioned fear extinction learning, a prefrontal cortex (PFC)-dependent process, as well as dysregulation of circadian and glucocorticoid hormone function. We examined the role of glucocorticoid-entrained clock gene expression within the PFC on conditioned fear extinction learning, and its interaction with glucocorticoid hormone profiles. We found a prominent time-of-day difference in extinction recall marked by superior recall in rats trained and tested at night. This superior recall required normal PFC clock gene expression as well as a combination of a diurnal pattern of circulating glucocorticoids and an acute elevation of glucocorticoids during the extinction learning memory consolidation period. Appropriate alignment of these factors in the clinical setting may enhance extinction learning related outcomes.

## Introduction

Conditioned fear learning, or the association of dangerous events (unconditioned stimulus; US) with associated environmental cues (conditioned stimulus; CS) is highly adaptive and is necessary for successful coping with future threats. However, the ability to inhibit a conditioned fear response once the CS no longer predicts danger is also of vital importance. Impaired extinction learning can lead to persistent anxiety and exaggerated fear that ultimately precipitates and exacerbates certain psychopathological conditions such as post-traumatic stress disorder (PTSD; Garfinkel et al., 2014). Exposure therapy is analogous to extinction learning in rodents (Milad et al., 2014), and it is one of the most frequently used techniques for treatment of PTSD (Craske et al., 2008). Unfortunately, partial or negligible response to treatment occurs in 20–60% of PTSD patients (van Minnen et al., 2002; Schottenbauer et al., 2008; Craske et al., 2014), emphasizing the need for research in this area. Further understanding of neuromechanisms that can enhance conditioned fear extinction learning may have valuable clinical applications.

Although conditioned fear learning primarily depends on local neural circuit adaptation within the amygdala, conditioned fear extinction learning and its recall also depends on the ventromedial prefrontal cortex [vmPFC; especially the infralimbic (IL) cortex; Sotres-Bayon et al., 2006; Sotres-Bayon and Quirk, 2010; Giustino and Maren, 2015]. PTSD is associated with dysregulation of various aspects of PFC function, and this dysregulation is believed to contribute to the impaired extinction learning present in some of these individuals (Garfinkel et al., 2014; Vanelzakker et al., 2014). PTSD is also associated with dysregulation of the circadian system and the hypothalamic-pituitary-adrenal (HPA) axis neuroendocrine system (Morris et al., 2012; Landgraf et al., 2014), two functionally interrelated major biological systems of the body (Dickmeis et al., 2013; Spencer et al., 2018). We have examined in rats the possible interaction of the circadian and HPA axis systems with vmPFC function and its role in the control of auditory conditioned fear extinction memory. In previous studies we found that the vmPFC has rhythmic 24-h clock gene (*Per1*, *Per2*, and *Bmal1*) expression patterns, suggestive of an intrinsic operational molecular clock (Chun et al., 2015). Moreover, we have found that normal vmPFC clock gene expression requires an appropriately-timed daily entraining pulse of circulating corticosterone (CORT), the principal effector hormone of the rat HPA axis (Woodruff et al., 2016). We and others also found that rats trained and tested during the nighttime (active phase) have superior conditioned fear extinction memory compared to rats trained and tested during the daytime (inactive phase; Chaudhury and Colwell, 2002; Woodruff et al., 2015). In addition, we have found that this superior nighttime extinction memory is absent in adrenalectomized (ADX) rats that lack circulating levels of CORT (Woodruff et al., 2015). These results led us to hypothesize that: (1) conditioned fear extinction learning is regulated in a circadian fashion, and (2) the time-of-day-dependent superior conditioned fear extinction learning and recall depends on a CORT entrained molecular clock within the vmPFC. To directly test whether a molecular clock in the vmPFC regulates conditioned fear extinction learning, we used a viral vector strategy to knock down *Per1* and *Per2* clock gene expression selectively in the vmPFC. We also tested whether a diurnal pattern of CORT secretion, which is necessary for normal PFC clock gene expression (Woodruff et al., 2016), would be sufficient to restore superior nighttime extinction learning in ADX rats. We found that superior extinction learning requires a combination of normal clock gene expression in the vmPFC, a diurnal pattern of circulating CORT, and an acute elevation of CORT during the memory consolidation period immediately after extinction training. Taken together, these findings illustrate a key role of the vmPFC molecular clock in coordination with circadian and training-induced elevations in CORT to strongly modulate auditory conditioned fear extinction learning.

## Materials and Methods

### Rats

Male Sprague Dawley rats (250–280 g at time of experimentation) were obtained from a commercial vendor (Envigo) and were maintained in animal facilities at the University of Colorado Boulder. Rats were housed two per cage (polycarbonate tubs, 47 × 23 × 20 cm) in a suite of rooms (each with independent control of light/dark cycle) that included the behavioral testing rooms. All rats were maintained on a 12/12 h light/dark cycle and were given two weeks to acclimate to the facility and light/dark cycle before behavioral testing. All procedures were conducted in accordance with the ethical treatment of animals and were approved by the University of Colorado Institutional Animal Care and Use Committee.

### Viral vector mediated knock-down of *Per1*/*Per2* expression in vmPFC

For knock-down of *Per1* and *Per2* clock gene expression in the vmPFC an adeno-associated virus (AAV2-Per1/Per2 shRNA+eGfp; Pol III U6 promoter) was used that transmits expression of a shRNA sequence directed against a conserved 24-base sequence (5′-ATCCCTCCTGACAAGAGGATCTTC-3′) within the coding region of the mouse/rat *Per1* and *Per2* genes. This virus also transmits expression of the enhanced green fluorescent protein (eGfp) reporter gene. For control comparisons, a similarly designed AAV virus (AAV-scrambled+eGfp) was used in which the shRNA encoding transgene was replaced with one that encodes a random sequence of 24 bases (5′-CGGAATTTAGTTACGGGGATCCAC-3′) with no known mammalian gene targets. The generation and validation of these viruses *in vitro* and *in vivo* in mouse brain have been previously described (Spencer et al., 2013).

**Figure 1.**
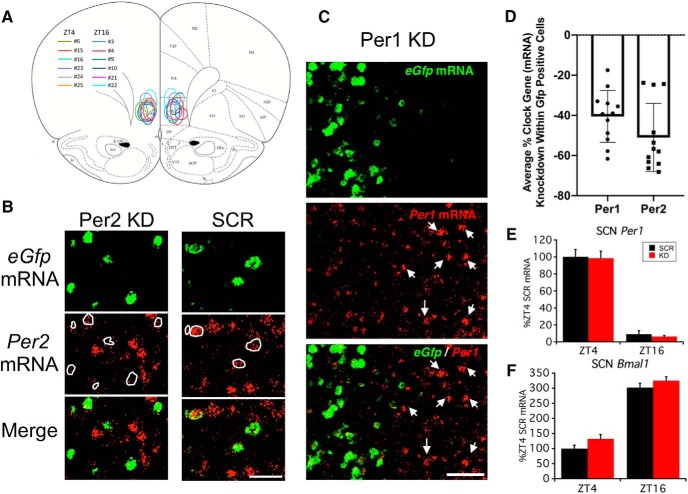
Selective knock-down of *Per1* and *Per2* mRNA within the vmPFC of rats in experiment 1. ***A***, Coronal brain atlas diagram (Paxinos and Watson, 1986) showing approximate location of AAV-Per1/Per2 shRNA+eGfp-infected cells (*eGfp* mRNA expression) for each individual rat in experiment 1 (key shows rat ID# and associated color outline). ***B***, Representative photomicrographs of relative *Per2* mRNA (red channel) immunofluorescence in virus-infected cells (*eGfp* mRNA, green channel) and nearby uninfected cells of a rat receiving the knock-down virus (KD) or control virus (SCR). Note the limited *Per2* mRNA expression in *eGfp* mRNA-positive cells (white outline) of KD but not SCR condition. Scale bar = 50 µm. ***C***, Representative photomicrograph of relative *Per1* mRNA (red channel) immunofluorescence in virus-infected cells (*eGfp* mRNA, green channel) and nearby uninfected cells (e.g., white arrows) of rat receiving the KD virus. Note the reduced immunofluorescence of *Per1* mRNA within the zone of infected cells. Scale bar = 50 µm. ***D***, Average percentage reduction of *Per1* and *Per2* mRNA immunofluorescence in *eGfp* mRNA-positive cells compared to nearby uninfected cells (*n* = 12 rats). ***E***, ***F***, SCN clock gene expression (*Per1* and *Bmal1* mRNA) of rats with vmPFC Per1/Per2 knock-down in experiment 1. Note, *Bmal1* and *Per1* mRNA represent positive and negative arm components of the molecular clock, respectively, and have expected antiphasic expression patterns (Chun et al., 2015) in rats regardless of AAV treatment condition. ***D–F*** depict mean ± SEM.

Rats under halothane anesthesia were given bilateral stereotaxic microinjection of 0.5-µl purified high titer virus directed toward the IL region of the PFC (Fig. 1*A*), using stereotaxic coordinates derived from Paxinos and Watson rat brain atlas (Paxinos and Watson, 1986; +3.2 mm A/P from bregma, ±0.5 mm M/L, –5.0 mm D/V). Microinfusions were delivered via a 10-µl Hamilton syringe with an attached 31 Ga beveled needle (Hamilton), with a delivery rate of 0.1 µl/min. The site and extent of viral infection was assessed in postmortem brains by examination of *eGfp* mRNA expression (see double-label fluorescent *in situ* hybridization procedure below).

### Experimental design and procedures

The interaction between time of day, PFC clock gene expression, and CORT condition on auditory conditioned fear learning and extinction learning was tested in three separate experiments. Time of day is expressed as Zeitgeber time (ZT; hours after light phase onset). Behavioral tests took place centered around the rats’ inactive phase (ZT4) or active phase (ZT16).

#### Experiment 1: test of vmPFC Per1/Per2 expression knock-down on conditioned fear/extinction learning

Rats were randomly assigned to one of four treatment conditions that resulted from the factorial combination of two between group factors (2 × 2 design): (1) time of day for training and testing (ZT4 or ZT16), and (2) AAV microinfusion into vmPFC (AAV-Per1/Per2 shRNA+eGfp or AAV-scrambled+Gfp); *n* = 5–6 per treatment condition. One week after virus microinfusion, rats were trained and tested on the 3-d auditory conditioned fear/extinction protocol as described below. Three days later, rats were killed by guillotine decapitation at the same time of day during which they had previously been trained and tested. Brains were rapidly extracted and frozen in an isopentane bath chilled to approximately –35°C. Brains were stored at –70°C until subsequent processing for clock gene and *eGfp* expression.

#### Experiment 2: test of the effect of ADX±diurnal CORT on conditioned fear/extinction learning

Rats were randomly assigned to one of six treatment conditions that resulted from the factorial combination of two between group factors (2 × 3 design): (1) time of day for training and testing (ZT4 or ZT16), and (2) CORT status (sham ADX, ADX, ADX+diurnal CORT); *n* = 6–8 per treatment condition. One week after surgery, rats were trained and tested on the 3-d auditory conditioned fear/extinction protocol as described below. Five days later, rats were killed by guillotine decapitation at the same time of day during which they had previously been trained and tested. Thymus glands were removed and weighed for determination of thymus wet weight relative to body weight as an indirect indicator of the extent to which CORT in the drinking water reestablished daily glucocorticoid levels within the normal physiologic range (Spencer and Deak, 2017).

#### Experiment 3: test of the effect of postextinction training acute CORT± diurnal CORT on extinction memory

Rats were randomly assigned to one of five CORT status conditions: (1) sham ADX, (2) ADX, (3) ADX+diurnal CORT, (4) ADX+acute CORT, and (5) ADX+acute and diurnal CORT; *n* = 7–8 per treatment condition. One week after surgery rats were trained and tested on the 3-d auditory conditioned fear/extinction protocol as described below. For this experiment all rats were trained and tested at ZT16. Fifteen minutes after the end of day 2 extinction training (during the glucocorticoid sensitive memory consolidation period; Roozendaal, 2002) rats were given an acute injection of CORT or vehicle. Five days after the day 3 extinction memory test rats were killed by guillotine decapitation at ZT16. Thymus glands were removed and weighed for determination of thymus wet weight relative to body weight.

### Conditioned fear/extinction training

Conditioned fear and extinction training/testing were administered as described previously (Woodruff et al., 2015). Each phase of the protocol can be conducted in a relatively short amount of time (<40 min), which permitted rats to be trained and tested at the same time each day. Training/testing at ZT4 was performed with the house lights on, and training/testing at ZT16 was performed under dim red lighting. For each session, freezing behavior (defined as lack of movement except for that due to respiration) was scored in real time by an experimenter blind to the treatment condition of each rat. Each session was video recorded, and behavioral scoring was verified by a second experimenter also blind to the treatment condition of each rat (∼90% inter-rater reliability). For the duration of each session rats were assessed every 10 s for the presence of freezing. A brief description of each session is provided below.

#### Day 1 acquisition (context A)

Conditioned fear acquisition took place in rectangular chambers (25.4 × 25.4 × 30.4 cm) comprised of three stainless steel walls and a Plexiglas front with a shock grid floor (stainless steel rods placed 2 mm apart). Rats were placed in context A for a 5-min pre-exposure period after which they were exposed to a single fear conditioning trial that consisted of presentation of a tone (30-s 1-kHz 70-dB tone; Coulbourn Instruments Audio Speaker) that coterminated with a single foot shock (2 s 0.8 mA; Coulbourn Precision Regulated Animal Shocker). Rats remained in the test chambers for 1 min postshock.

#### Day 2 extinction (context B)

Conditioned fear recall testing and extinction training took place in clear Plexiglas boxes (28.6 × 18.5 × 22 cm) with wire mesh floors. These boxes were contained within sound attenuating icebox coolers containing the same type of audio speakers used in context A. Context B boxes, but not context A boxes, were cleaned with 70% ethanol and allowed to dry between each session. Context B boxes were scented with peppermint oil (Now Foods). On day 2 (24 h after day 1 acquisition), rats were pre-exposed to context B for 3 min followed by 15 trials of CS alone (30-s 1-kHz 70-dB tone unaccompanied by a foot shock) with a random intertrial interval (ITI) of 90–120 s. For ease of viewing and analysis, freezing data for this session were organized into blocks of three (tone+ITI) for a total of five trial blocks. Freezing behavior during the first trial block was used as a measurement of 24-h conditioned fear recall.

#### Day 3 extinction recall (context B)

On day 3, rats were returned to context B and presented with six additional trials of CS alone, using the same parameters as on day 2. For ease of viewing and analysis, freezing data for this session were organized into blocks of two trials for a total of three trial blocks.

### CORT manipulations

For ADX, rats were anesthetized with halothane, and bilateral incisions were made through the dorsal lateral skin and peritoneal wall near the kidney. Adrenal glands of ADX rats were isolated with forceps and excised. Sham rats went through the same procedure, but adrenal glands were left in place. ADX rats were maintained on 0.9% saline drinking water ± CORT (25 µg/ml). The CORT (Steraloids) added to the drinking water was first dissolved in ethanol resulting in a final concentration of 25 µg/ml CORT and a low level of 0.1% ethanol. This dosage/treatment has been shown to produce circulating plasma CORT levels that closely mimic the levels and pattern of diurnal CORT circulation observed in adrenal-intact male rats (Jacobson et al., 1988; Malek et al., 2007). Sham or ADX rats not given CORT in the drinking water were given a matched level of ethanol (0.1% ethanol) in their drinking water. The assigned diurnal CORT treatment began immediately after surgery and was maintained for the remainder of the experiment. In experiment 3, rats received an acute injection of either vehicle (60% sterile saline, 30% propylene glycol, 10% ethanol; 1 ml/kg; i.p.) or CORT (2.5 mg/kg; i.p.) 15 min after the end of day 2 extinction training. This CORT treatment produces peak plasma CORT levels within 30 min after injection (∼40–50 µg/100 ml) that are similar to those observed in adrenal-intact rats in response to acute stress (Spencer and Deak, 2017).

### *In situ* hybridization and image analysis

Coronal brain slices (12 µm) were cut on a Leica Microsystems cryostat (model 1850) extending from the beginning of the PFC (+4.2 mm anterior to bregma) through the caudal extent of the suprachiasmatic nucleus (SCN; –1.4 mm posterior to bregma; Paxinos and Watson, 1986), thaw mounted on Colorfrost Plus slides (VWR), and stored at –70°C.

#### Single radiolabeled *in situ* hybridization

Single radiolabeled *in situ* hybridization for *Per1* and *Bmal1* mRNA expression in the SCN was performed as previously described (Girotti et al., 2009). Briefly, slides were fixed in a buffered 4% paraformaldehyde mixture for 15–30 min followed by a 2× saline-sodium citrate solution (SSC), acetylated for 10 min (0.1 M triethanolamine, acetic anhydride; pH 8), rinsed in RNase-free water, and dehydrated in a series of increasing ethanol solutions. Tissue was then hybridized with ^35^S-UTP-labeled antisense riboprobes in a 50% formamide humidified atmosphere at 54°C for 16–18 h, after which slides were treated with RNase A (Sigma) at 37°C for 1 h, washed in decreasing concentrations of SSC (2×, 1×, 0.5×, 0.1×), incubated in a high stringency wash in 0.1× SSC at 65°C for 1 h, then dehydrated through a series of ethanol washes. Dried slides were then exposed to x-ray film (Kodak Biomax XAR) for two weeks. The coding portion of the *Per1* and *Bmal1* genes used for riboprobe generation is: *Per1* nuclear transcript 974–1547, GenBank accession no. NM_001034125, and *Bmal1* nuclear transcript 697–1278, GenBank accession no. NM_024362.

Autoradiograph images were digitized by use of a lightbox (Northern Light model B95; Imaging Res. Inc), a CCD video camera (Sony model XC-ST70) fitted with a Navitar 7000 zoom lens connected to a LG3-01 frame grabber (Scion Corp.) inside a Dell Dimension 500 computer, and captured with Scion Image beta software (release 4.0.2). Densitometry measurements for the SCN were taken from left and right hemispheres on four sections per brain by an individual blind to treatment condition. Measurements taken were 12 pixels × 12 pixels in size and were centered over the target brain region as outlined in a rat brain atlas (Paxinos and Watson, 1986). Densitometry measurements (uncalibrated optical density) were performed using ImageJ software 1.46r (National Institutes of Health). Optical density values were averaged across each of the separate tissue sections/hemisphere measures for each brain to yield a single value per rat.

#### Double label fluorescent *in situ* hybridization

For double label (*Per1* or *Per2* and *eGfp* mRNA) fluorescent-tagged *in situ* hybridization, riboprobes were generated as described above, but either digoxigenin-labeled UTP (for *Per1* or *Per2*) or fluorescein-labeled UTP (for *eGfp*) was incorporated into the antisense riboprobe. Initial fixation, hybridization, and posthybridization procedures were conducted as described for single radiolabeled *in situ* hybridization until completion of the high stringency wash, after which slides were transferred to 0.05 M sodium PBS and stored overnight at 4°C overnight. The next day, slides were washed in PBS and then endogenous peroxidase was quenched by incubation in phosphate-buffered 2% hydrogen peroxide (30 min). Slides were then washed in 0.1 M Tris-buffered saline (TBS) with 0.05% Tween 20 (TBS-T) followed by 0.5% Blocking reagent (PerkinElmer) in TBS. For detection of *Per1/Per2* mRNA, slides were exposed to horseradish-peroxidase linked anti-digoxigenin antibody (Roche Molecular Systems, Inc.) at 1:750 in 0.5% Blocking reagent for 30 min, then washed with 0.1 M TBS. Amplification of the mRNA signal with a fluorescent marker was performed using the Cy3 tyramide amplification reagent (PerkinElmer) according to manufacturer’s directions. Slides were washed with 0.1 M TBS-T, and the process was repeated for detecting the *eGfp* mRNA using anti-fluorescein-HRP (1:100; PerkinElmer) and the Fluorescein tyramide amplification reagent (PerkinElmer). Slides were rinsed with PBS, incubated with DAPI nuclear stain at 1:30,000 (Fisher Scientific) and then rinsed again. Wet slides were coverslipped with mounting media (Fluoromount; Southern Biotech or Prolong Gold; Invitrogen). Proper injection site placement, AAV infection (expression of *eGfp* mRNA), and *Per1* and *Per2* knock-down (expression of *Per1/Per2* mRNA in area of *eGfp* expression relative to *Per1/Per2* adjacent expression) were verified on brain slices at the level of the PFC (Paxinos and Watson, 1986) by assessing relative riboprobe immunofluorescence using a Zeiss Axioimager M1 microscope with supporting Zeiss Axiovision image analysis software for image capture (Carl Zeiss Microscopy GmbH), followed by quantification of relative immunofluorescence intensity using ImageJ software 1.46r. Relative *Per1* or *Per2* mRNA immunofluorescence was measured within *eGfp* mRNA-positive cells and adjacent *eGfp* mRNA negative cells (∼100 cells each per injection site). Relative immunofluorescence was measured in both hemispheres of one to two sections per brain and then the percentage decrease within each injection site averaged for each brain. The location and extent of viral mediated *eGfp* mRNA expression was determined for each brain by examining a series of rostral-caudal coronal sections throughout the PFC and drawing the relative location, shape and size of expression as viewed through the microscope onto printouts of relevant rat brain atlas diagrams (Paxinos and Watson, 1986). The medial-lateral and dorsal-ventral diameter of *eGfp* expression was measured on brain images captured at the level of maximal expression using Axiovision software measurement tools.

### Statistical analysis

Mixed design ANOVA was used to test for within and between group treatment effects on freezing behavior for each training/test day. For experiment 1, time of day and virus condition were between group factors; for experiment 2, time of day and CORT status were between groups factors, and for experiment 3, CORT status was a between group factor. For each experiment, trial or trial block was a within group factor. In the case of significant ANOVA main effects and interactions, Fisher’s least significant difference (FLSD) was used as a *post hoc* test for between group differences within a specific trial or trial block. For training/test days 2 and 3, pre-tone freezing was analyzed separately and was not included in the trial block by treatment group ANOVAs. The statistical package for the social sciences (SPSS, version 3.0 1992) was used for these analyses; *p* < 0.05 was considered significant. Data presented in figures are the mean ± SEM, and all statistically significant effects are reported.

## Results

### Experiment 1. Knock-down of *Per1*/*Per2* mRNA in vmPFC prevents time-of-day-dependent superior conditioned fear extinction recall

#### Postmortem assessment of viral expression and *Per1/Per2* mRNA knock-down

Strong viral infection (*eGfp* mRNA expression) was observed in all brains that received the AAV-Per1/Per2 shRNA+eGfp microinfusion. The infection was primarily centered within the IL, and the infection zone was generally spherical or ovoid (with a longer axis in the dorsal-ventral dimension; Fig. 1*A*). The average (±SD) diameter of the infection zone, as measured on a coronal section near the center of maximal *eGfp* expression for each brain, was 757 ± 108 µm medial-lateral and 1162 ± 266 µm dorsal-ventral. Because brains showed viral infection that was not always restricted to the IL, but in some cases also included ventral and rostral prelimbic (PL) cortex, we refer throughout this manuscript to the site of viral vector manipulation as the vmPFC. There was an average 40% and 50% reduction of *Per1* and *Per2* mRNA immunofluorescence, respectively, in infected cells (*Gfp* mRNA-positive cells) compared to nearby uninfected cells within the vmPFC (Fig. 1*B–D*). Within the SCN there was no effect of AAV-Per1/Per2 shRNA status on the expected time-of-day difference in *Per1* (Fig. 1*E*) and *Bmal1* (Fig. 1*F*) mRNA expression (Chun et al., 2015), indicating that this treatment did not affect the SCN Master Clock.

#### Conditioned fear acquisition, extinction, and extinction recall

**Figure 2.**
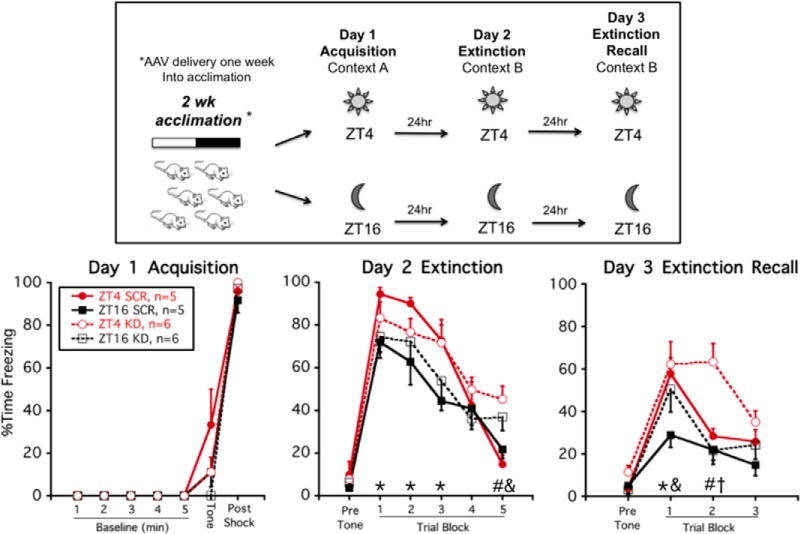
Knock-down of *Per1*/*Per2* mRNA in vmPFC prevents time-of-day-dependent (ZT16) superior conditioned fear extinction recall. Experimental timeline (upper panel): rats received bilateral microinfusion of AAV-Per1/Per2 shRNA+eGfp (KD) or control virus (SCR) in vmPFC and one week later were trained and tested in a 3-d auditory conditioned fear/extinction regimen at either ZT4 or ZT16. Freezing behavior (lower panel, mean ± SEM): there was no overall effect of time of day on day 1 acquisition, or 24-h conditioned fear recall (day 2, trial block 1), and no time of day by virus condition interaction for within session extinction (day 2, trial blocks 2–5). But rats given control virus had superior extinction recall at ZT16 compared to ZT4 (day 3, trial block 1). Knock-down of *Per1*/*Per2* mRNA within the vmPFC prevented this superior extinction recall; *p < 0.05, ZT4 SCR versus ZT16 SCR (FLSD); ^#^*p* < 0.05, ZT4 SCR versus ZT4 KD (FLSD), ^&^*p* < 0.05, ZT16 SCR versus ZT16 KD (FLSD); ^†^*p* < 0.05, ZT4 KD versus ZT16 KD (FLSD).

As we have previously demonstrated (Woodruff et al., 2015), on day 1 (acquisition) there was no effect of time of day on the acquisition of conditioned fear (Fig. 2). There was also no effect of virus condition on day 1 freezing levels. Rats trained at either ZT4 or ZT16 exhibited minimal freezing before the tone-shock pairing and displayed high freezing during the 1-min postshock observation period (within subjects effect of session phase, *F*_(6,84)_ = 223.6; *p* < 0.001).

On day 2 (extinction training), all rats exhibited a high degree of freezing during the initial block of three trials, indicating that all rats had strong 24-h recall of auditory conditioned fear. All groups of rats also displayed a progressive decline in freezing across the session (significant within subject effect of trial block, *F*_(4,68)_ = 45.9; *p* < 0.001; Fig. 2). There was no within subject trial block interaction with time of day or virus condition, indicating that all rats extinguished conditioned fear at a similar rate. There was a significant between subjects effect of time of day (*F*_(1,17)_ = 5.0; *p* = 0.04), such that ZT4 rats generally exhibited higher freezing levels than ZT16 rats during extinction training. *Post hoc* analysis indicated that ZT16 SCR rats exhibited less freezing than ZT4 SCR rats during the first three trial blocks (*p* < 0.05, FLSD). During the final trial block, control virus (SCR)-treated rats at both times of day exhibited less freezing than knock-down virus (KD)-treated rats (*p* < 0.05, FLSD).

On day 3 (extinction recall), SCR rats trained and tested at ZT16 exhibited superior extinction recall compared to SCR rats trained and tested at ZT4 (between subjects effect of time-of-day: *F*_(1,17)_ = 8.8; *p* = 0.009; Fig. 2). This result is consistent with previous findings (Chaudhury and Colwell, 2002; Woodruff et al., 2015). *Post hoc* analysis indicated that during the first trial block ZT16 SCR rats exhibited less freezing than ZT4 SCR rats (*p* < 0.05). This time-of-day effect was absent in ZT16 KD rats whose freezing levels were comparable to that of ZT4 SCR and ZT4 KD rats. Thus, knock-down of clock gene expression selectively within the vmPFC was sufficient to prevent the superior conditioned fear extinction recall normally seen in rats trained and tested during their active phase (ZT16). During trial block 2, ZT4 KD rats exhibited higher freezing than all other rats, suggesting that this treatment group had some greater resistance to extinction than the other groups (between subjects effect of viral condition: *F*_(1,17)_ = 8.4; *p* = 0.01; within subject trial block by viral condition by time-of-day interaction: *F*_(2,34)_ = 5.6; *p* = 0.008).

### Experiment 2. ADX prevents time-of-day-dependent superior conditioned fear extinction recall; however, a diurnal pattern of CORT exposure is not sufficient to restore superior recall in ADX rats

The ability of *Per1*/*Per2* knock-down in the vmPFC to selectively impair auditory conditioned fear extinction recall in rats trained and tested at ZT16 is similar to the effect of ADX that we have previously observed on these measures (Woodruff et al., 2015). It is possible that the impaired extinction recall of ADX rats trained and tested at ZT16 is due to the disrupted PFC rhythmic clock gene expression observed in ADX rats (Woodruff et al., 2016). Providing ADX rats with a diurnal pattern of daily CORT exposure is sufficient to normalize disrupted PFC rhythmic clock gene expression in ADX rats (Woodruff et al., 2016). Therefore, in this second experiment we tested whether giving ADX rats a diurnal pattern of circulating CORT (ADX+dCORT) would restore superior conditioned fear extinction recall in ADX rats trained and tested at ZT16.

**Figure 3.**
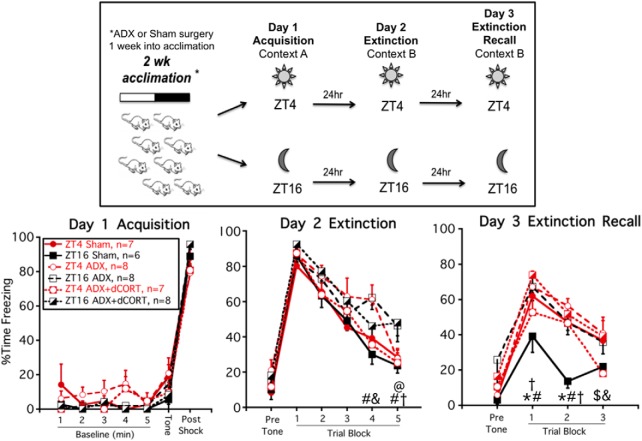
ADX ± diurnal CORT replacement prevents time-of-day-dependent conditioned fear extinction recall. Experimental timeline (upper panel): rats were given sham or ADX surgery one week before conditioned fear/extinction training. Some ADX rats were also given CORT in their drinking water to restore a diurnal pattern of circulating CORT (ADX+dCORT). Rats were trained and tested at either ZT4 or ZT16. Freezing behavior (lower panel, mean ± SEM): there was no overall effect of time of day or adrenal status on day 1 acquisition, 24-h conditioned fear recall (day 2, trial block 1) or within session extinction (day 2, trial blocks 2–5). But sham rats trained and tested at ZT16 had superior extinction recall compared to each of the other groups (day 3, trial blocks 1 and 2); *p < 0.05, ZT4 sham versus ZT16 sham (FLSD); ^#^*p* < 0.05, ZT16 sham versus ZT16 ADX (FLSD); ^&^*p* < 0.05, ZT4 ADX versus ZT4 ADX+dCORT (FLSD); ^@^*p* < 0.05, ZT4 ADX+dCORT versus ZT16 ADX+dCORT (FLSD); ^†^*p* < 0.05, ZT16 sham versus ZT16 ADX+dCORT (FLSD); ^$^*p* < 0.05, ZT4 sham versus ZT4 ADX+dCORT.

There was no effect of time of day or ADX on day 1 acquisition of conditioned fear (Fig. 3). On day 2 (extinction training), all rats exhibited a high degree of freezing during the initial block of three trials, indicating that each group of rats had a comparably high level of 24-h recall of auditory conditioned fear. All groups of rats also exhibited progressively lower freezing over the course of the session (within subject effect of trial block; *F*_(4148)_ = 86.8; *p* < 0.001). There was also a significant within subject trial block by CORT status interaction (*F*_(8148)_ = 1.4; *p* = 0.03), due primarily to some between group variation in freezing levels across the last two trial blocks. However, there were no overall between-subjects effects of time-of-day or adrenal status, suggesting similar levels of within session extinction learning.

On day 3 (extinction recall) sham ZT16 rats exhibited superior extinction recall compared to sham ZT4 rats, and this time-of-day effect was absent in ADX rats (between subjects time of day by CORT status interaction: *F*_(2,37)_ = 5.6; *p* = 0.008; Fig. 3). Sham ZT16 rats had less freezing than all other groups of rats during the second trial block, and less freezing than all other groups except ADX+dCORT ZT4 rats during the first trial block (*p* < 0.05, FLSD). These results corroborate our previous findings (Woodruff et al., 2015). In this experiment, we also found that diurnal CORT replacement was not able to restore superior extinction recall in ADX rats trained and tested at ZT16.

There was an overall effect of CORT status on thymus weight (CORT status: *F*_(2,41)_ = 21.1; *p* < 0.001). As expected (Spencer and Deak, 2017), thymus weight relative to body weight in ADX rats (190.4 ± 6.7 mg/100 g; mean ± SEM) was higher than that in Sham rats (139.3 ± 6.2 mg/100 g; mean ± SEM). CORT replacement in ADX rats (134.7 ± 7.6 mg/100 g; mean ± SEM) normalized thymus weights (*p* < 0.05, FLSD), indicating that the diurnal CORT treatment was able to restore circulating CORT levels to within the normal physiologic range.

### Experiment 3. A diurnal pattern of CORT exposure combined with postextinction training CORT elevation is sufficient to restore superior conditioned fear extinction recall in ADX rats

Although giving ADX rats CORT in their drinking water restores a circadian-like pattern of circulating CORT levels, those rats lack the ability to mount an acute CORT response to stressful situations. A number of studies find that elevated glucocorticoid levels present immediately after emotional learning tasks facilitate memory consolidation and subsequent memory recall (de Quervain et al., 2009). Therefore, the superior conditioned fear extinction recall that we observe in adrenal-intact rats trained and tested at ZT16 may require both a circadian pattern of circulating CORT as well as an acute elevation of CORT during the post extinction training consolidation period. This third experiment tested this prospect. In addition to the three CORT status conditions examined in experiment 2 (sham, ADX, ADX+dCORT) this experiment also included two more groups of ADX rats that received an acute injection of CORT (aCORT) 15 min after day 2 extinction training with or without CORT in the drinking water. Because we do not see superior extinction recall in adrenal-intact rats trained and tested at ZT4, this experiment only examined the effect of CORT status on rats trained and tested at ZT16.

As we demonstrated in experiment 2, there was no effect of ADX ± diurnal CORT replacement on conditioned fear acquisition or the 24-h recall of conditioned fear. On day 2, there was a significant within subject trial block by CORT status interaction (*F*_(4124)_ = 3.4; *p* = 0.002), and a significant between subjects effect of CORT status (*F*_(1,33)_ = 7.8; *p* = 0.002), reflecting the fact that ADX rats exhibited a somewhat slower rate of extinction compared to sham and ADX+dCORT-treated rats. *Post hoc* analysis indicates that during trial blocks 3 and 4, ADX rats exhibited higher freezing behavior than both sham and ADX+dCORT-treated rats. There was no difference between groups for the final trial block.

**Figure 4.**
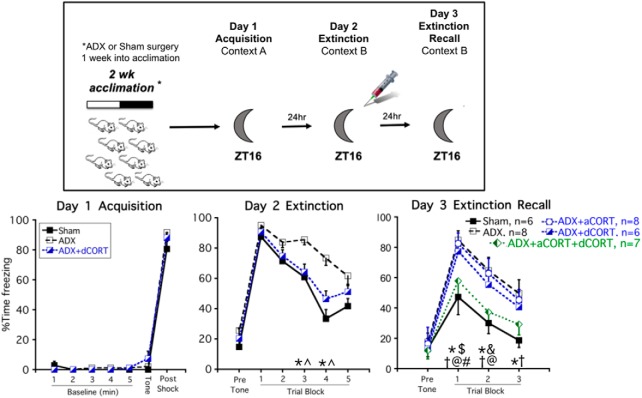
A combination of diurnal CORT and post extinction training acute CORT restores superior conditioned fear extinction recall in ADX rats. Experimental timeline (upper panel): rats were given sham or ADX surgery one week before conditioned fear/extinction training. Some ADX rats were also given CORT in their drinking water (ADX+dCORT), an acute injection of CORT immediately after day 2 extinction training (ADX+aCORT) or both (ADX+aCORT+dCORT). All rats were trained and tested at ZT16. Freezing behavior (lower panel, mean ± SEM): there was no overall effect of adrenal status on day 1 acquisition, 24-h conditioned fear recall (day 2, trial block 1) or within session extinction (day 2, trial blocks 2–5). But sham and ADX+aCORT+dCORT rats had superior extinction recall compared to each of the other groups (day 3, trial blocks 1 and 2). Data for ADX rats that had not yet received an acute injection of CORT or saline after day 2, were pooled on days 1 and 2 for clarity of presentation; *p < 0.05, sham versus ADX (FLSD); ^^^*p* < 0.05, ADX versus ADX+dCORT (FLSD); ^#^*p* < 0.05, sham versus ADX+dCORT (FLSD); ^†^*p* < 0.05, sham versus ADX+aCORT (FLSD); ^@^*p* < 0.05, ADX+aCORT+dCORT versus ADX (FLSD); ^$^*p* < 0.05, ADX+aCORT+dCORT versus ADX+dCORT (FLSD); ^&^*p* < 0.05, ADX+aCORT+dCORT versus ADX+aCORT (FLSD).

On day 3 (extinction recall) ADX produced a deficit in extinction recall compared to sham rats, which is consistent with experiment 2 and our previous report (Woodruff et al., 2015). Neither acute CORT (ADX+aCORT) nor diurnal CORT (ADX+dCORT) treatment of ADX rats was sufficient to prevent this deficit in extinction recall (Fig. 4). Importantly, ADX rats that received both acute and diurnal CORT replacement (ADX+aCORT+dCORT) exhibited extinction recall that was similar to sham rats. There was an overall between subjects effect of CORT status (*F*_(1,30)_ = 5.1; *p* = 0.003), and *post hoc* tests confirm that there was no difference between the sham and ADX+aCORT+dCORT groups for each trial block, whereas both groups differed from the ADX group for each trial block (Fig. 4). Thus, giving ADX rats the combination of a diurnal pattern of circulating CORT and an acute elevation of CORT during the postextinction training consolidation period was able to restore the superior extinction recall of rats trained and tested at ZT16.

Similar to experiment 2, there was an overall effect of CORT status on thymus weight (CORT status: *F*_(4,40)_ = 6.1; *p* = 0.001). ADX rats exhibited thymic hypertrophy (232.1 ± 8.8; mean ± SEM) compared to Sham rats (183.6 ± 12.4; mean ± SEM). This was normalized in ADX rats given CORT in the drinking water (ADX+dCORT: 177.2 ± 7.2; ADX+aCORT+dCORT: 165.1 ± 13.3; mean ± SEM), but not in ADX rats given only an acute injection of CORT (ADX+aCORT: 207.6 ± 11.1; mean ± SEM), again indicating that the CORT in the drinking water treatment resulted in circulating CORT levels within the normal physiologic range.

## Discussion

This study demonstrates that the time of day during which rats are trained and tested results in a large difference in auditory conditioned fear extinction learning. Rats trained and tested during the night (active phase) have superior 24-h extinction recall compared to rats trained and tested during the day (inactive phase). This finding is consistent with previous reports of time-of-day differences in extinction learning in mice, rats and humans (Chaudhury and Colwell, 2002; Pace-Schott et al., 2013; Woodruff et al., 2015). Remarkably, virally mediated knock-down of *Per1* and *Per2* clock gene expression in the vmPFC was sufficient to eliminate this nighttime superior extinction learning. In addition, the nighttime superior extinction recall required a combination of both a diurnal pattern of circulating CORT and an acute elevation of CORT during the postextinction training memory consolidation period. These results support the prospect that the superior conditioned fear extinction learning depends on a CORT entrained molecular clock in the vmPFC. Moreover, this molecular clock may regulate in a circadian fashion the neuroplasticity processes necessary for optimal extinction learning that includes the enhancing influence of acute CORT during memory consolidation.

It is noteworthy that the one aspect of conditioned fear expression that is strongly modulated by the vmPFC, extinction recall (Knapska and Maren, 2009; Do-Monte et al., 2015; Marek et al., 2018), was the aspect that showed a pronounced time-of-day dependence. Furthermore, we found that disruption of vmPFC clock gene expression and diurnal CORT circulation prevented the time-of-day effect on conditioned fear extinction learning, while having no effect on the development and recall of conditioned fear. These findings suggest an interdependence of time of day, vmPFC clock gene expression, and diurnal CORT in modulating vmPFC control of conditioned fear extinction learning. Intriguingly, this modulation was primarily restricted to the superior extinction learning of rats trained and tested during their active phase.

Our results suggest that auditory conditioned fear extinction learning is regulated in a circadian fashion. Further support for this possibility is that two different strains of mice maintained in constant darkness also displayed superior conditioned fear extinction recall when trained and tested during their circadian subjective night compared to their circadian subjective day (Chaudhury and Colwell, 2002). In addition, our results support the hypothesis that this circadian regulation of conditioned fear extinction learning depends on a molecular clock operational within PFC neurons. Although oscillatory clock gene expression has been found in a number of extra-SCN brain regions, characterization of the functional relevance of this clock gene expression is limited. Several studies, however, have demonstrated that selective disruption of clock gene expression in extra-SCN brain regions results in altered behavioral and cognitive processes (Mukherjee et al., 2010; Spencer et al., 2013; Snider et al., 2016; Bering et al., 2018). This study extends these findings by demonstrating a functional role of normal clock gene expression in the vmPFC for regulation of auditory conditioned fear extinction learning. In this study there was no alteration of SCN clock gene expression in rats receiving vmPFC clock gene knock-down, indicating that the observed behavioral effects were a direct result of altered vmPFC clock gene expression, rather than altered SCN clock function.

We found that time of day did not affect auditory conditioned fear acquisition and recall. Other studies have also found a lack of diurnal modulation of auditory conditioned fear acquisition and recall (Rudy and Pugh, 1998; Hopkins and Bucci, 2010; Melo and Ehrlich, 2016), but for an exception see (Chaudhury and Colwell, 2002). In contrast, acquisition and recall of contextual conditioned fear, a hippocampal-dependent process, is modulated by time of day, with some (Chaudhury and Colwell, 2002; Eckel-Mahan et al., 2008; Snider et al., 2018), but not all studies (Hopkins and Bucci, 2010) observing superior contextual conditioned fear recall present in nocturnal rodents trained and tested during the day (inactive phase). There may be an adaptive advantage for circadian regulation of conditioned fear extinction learning. If rats encounter a direct threat, such as a predator attack during their inactive phase (when they should be safe in their burrow) then it may be important to be relatively resistant to extinction of associated threat cues at that time of day. In contrast, it may be advantageous to readily develop extinction to similar threat cues that occur during the active phase, when the rat must continue to pursue daily activities essential to life. Circadian regulation of brain function may be especially tuned to fear-related learning as there is recent evidence that daily fear exposure can serve as a circadian phase entrainment factor (Pellman et al., 2015).

An additional feature of our study concerns the requirement of endogenous CORT secretion for the nighttime-dependent superior conditioned fear extinction learning of rats. We reproduced in this study our previous finding that ADX prevents the superior nighttime extinction learning. One possibility for this loss of superior extinction learning is the impairment in PFC clock gene expression present in ADX rats (Woodruff et al., 2016). The proper circadian phase of clock gene expression in the PFC is believed to ultimately depend on daily entrainment signals originating from the body’s master clock, the SCN. The SCN, however, projects to a very limited number of brain regions, and it does not project directly to the PFC (Watts et al., 1987; Sylvester et al., 2002). One avenue by which the SCN relays daily entrainment information to tissue clocks located in the rest of the body is through the SCN-controlled circadian rhythm of CORT secretion (Spencer et al., 2018). ADX results in disrupted PFC clock gene expression, but normal expression profiles are restored in ADX rats given an appropriately timed daily systemic injection of CORT (Woodruff et al., 2016). Consequently, we tested in this study whether giving ADX rats a diurnal pattern of circulating CORT would be sufficient to restore nighttime superior extinction learning. We found, however, that although a diurnal pattern of circulating CORT was necessary for the nighttime superior extinction learning, it was not sufficient. Instead, ADX rats also required an acute elevation of CORT present immediately after extinction training. A number of studies have found that elevation of glucocorticoids during or immediately after various emotional experiences, including conditioned fear extinction learning, leads to enhanced memories associated with that experience (Pugh et al., 1997; Barrett and Gonzalez-Lima, 2004; Hui et al., 2004; de Quervain et al., 2009; Blundell et al., 2011).

It appears from our study that there is an interaction between circadian CORT secretion and acute elevations of CORT that specifically augments the memory consolidation process of a conditioned fear extinction training session. Some possible underlying mechanisms by which this interaction may occur includes circadian modulation of intracellular signal transduction and dendritic spine dynamics in the vmPFC. For example, several intracellular biochemical signaling factors and pathways, including ERK/MAPK, mTOR and CREB are implicated in PFC regulation of conditioned fear extinction learning (Lin et al., 2003; Hugues et al., 2004; Girgenti et al., 2017), and these same pathways are also responsive to circadian and glucocorticoid manipulations (Eckel-Mahan et al., 2008; Cao et al., 2011; Osterlund et al., 2011; Chen et al., 2012; Xiong et al., 2015; Coria-Lucero et al., 2016). In addition, each of these factors modulates dendritic spine density and morphology (Perez-Cruz et al., 2009; Liston et al., 2013; Lai et al., 2015; Anderson et al., 2016; Tuscher et al., 2016; Serita et al., 2017).

## Concluding Perspectives

We demonstrate in this study that optimal auditory conditioned fear extinction learning is reliant on the presence of normal clock gene expression in the vmPFC as well as the presence of both circadian and training-associated elevations of glucocorticoid hormone. These factors lead to superior extinction learning when rats are trained and tested at night, during their active phase. Interestingly, consistent with our rodent work, some human studies find better extinction learning and improved exposure therapy results when therapeutic sessions are conducted during the morning (human active phase) compared to the evening (Pace-Schott et al., 2013; Meuret et al., 2016; Zuj et al., 2016). Moreover, some research shows that glucocorticoid elevation during exposure therapy is beneficial in treating PTSD and phobias (Yehuda et al., 2015).

Circadian regulation of PFC function is likely to impact a number of different aspects of PFC function, of which conditioned fear extinction learning is just one example. In humans, a number of PFC-dependent cognitive processes exhibit diurnal variation (Babkoff et al., 2005; Matchock and Mordkoff, 2009; Mendoza and Challet, 2009; Marek et al., 2010; Soshi et al., 2010). Experimental disruption of circadian rhythms in mice leads to decreased medial PFC dendritic complexity and impaired medial PFC-dependent behaviors (Karatsoreos et al., 2011). Given the strong association between psychiatric disorders and altered PFC function (Arnsten, 2009), establishment of the proper entrainment and operation of the molecular clock within various subregions of the PFC may be a therapeutic goal that is beneficial in treating not only PTSD, but other psychiatric disorders. Finally, this study highlights the value of examining neurobehavioral processes during both the animal subject's inactive and active phase.

## Synthesis

Reviewing Editor: Carmen Sandi, Swiss Federal Institute of Technology Decisions are customarily a result of the Reviewing Editor and the peer reviewers coming together and discussing their recommendations until a consensus is reached. When revisions are invited, a fact-based synthesis statement explaining their decision and outlining what is needed to prepare a revision will be listed below. Note: If this manuscript was transferred from JNeurosci and a decision was made to accept the manuscript without peer review, a brief statement to this effect will instead be what is listed below.

Based on a reported dysregulation of the circadian rhythms in PTSD, and previous work suggesting a corticosterone-related circadian clock in the prefrontal cortex, this study investigated the involvement of vmPFC clock gene expression and associated corticosterone in the regulation of extinction of fear conditioning (animals tested either on the light or the dark phase of the circadian cycle). The authors report a series of comprehensive and elegant studies involving Per1 and Per2 gene knockdown, adrenalectomy and corticosterone replacement. The findings show a clear interaction between corticosterone levels and expression of clock genes in the vmPFC for the expression of fear extinction / recall. These novel observations may have relevant implications for PTSD coadjuvant treatments. The authors have addressed appropriately previous questions raised by former reviewers related to the characterization of the viral manipulations and clarification of several illustrations. The report constitutes an important addition to the literature helping to dissect key brain-periphery interactions on the control of behavior.

## References

[B1] Anderson RM, Glanz RM, Johnson SB, Miller MM, Romig-Martin SA, Radley JJ (2016) Prolonged corticosterone exposure induces dendritic spine remodeling and attrition in the rat medial prefrontal cortex. J Comp Neur 524:3729–3746. 10.1002/cne.24027 27113541 PMC5063662

[B2] Arnsten AFT (2009) Stress signalling pathways that impair prefrontal cortex structure and function. Nat Rev Neurosci 10:410–422. 10.1038/nrn2648 19455173 PMC2907136

[B3] Babkoff H, Zukerman G, Fostick L, Ben-Artzi E (2005) Effect of the diurnal rhythm and 24 h of sleep deprivation on dichotic temporal order judgment. J Sleep Res 14:7–15. 10.1111/j.1365-2869.2004.00423.x 15743328

[B4] Barrett D, Gonzalez-Lima F (2004) Behavioral effects of metyrapone on Pavlovian extinction. Neurosci Lett 371:91–96. 10.1016/j.neulet.2004.08.046 15519735

[B5] Bering T, Carstensen MB, Wörtwein G, Weikop P, Rath MF (2018) The circadian oscillator of the cerebral cortex: molecular, biochemical and behavioral effects of deleting the Arntl clock gene in cortical neurons. Cereb Cortex 28:644–657. 10.1093/cercor/bhw406 28052921

[B6] Blundell J, Blaiss CA, Lagace DC, Eisch AJ, Powell CM (2011) Block of glucocorticoid synthesis during re-activation inhibits extinction of an established fear memory. Neurobiol Learn Mem 95:453–460. 10.1016/j.nlm.2011.02.006 21333745 PMC3356929

[B7] Cao R, Anderson FE, Jung Y-J, Dziema H, Obrietan K (2011) Circadian regulation of mammalian target of rapamycin signaling in the mouse suprachiasmatic nucleus. Neuroscience 181:79–88. 10.1016/j.neuroscience.2011.03.005 21382453 PMC3102430

[B8] Chaudhury D, Colwell CS (2002) Circadian modulation of learning and memory in fear-conditioned mice. Behav Brain Res 133:95–108. 12048177 10.1016/s0166-4328(01)00471-5

[B9] Chen DY, Bambah-Mukku D, Pollonini G, Alberini CM (2012) Glucocorticoid receptors recruit the CaMKIIα-BDNF-CREB pathways to mediate memory consolidation. Nat Neurosci 15:1707–1714. 10.1038/nn.3266 23160045 PMC3509234

[B10] Chun LE, Woodruff ER, Morton S, Hinds LR, Spencer RL (2015) Variations in phase and amplitude of rhythmic clock gene expression across prefrontal cortex, hippocampus, amygdala, and hypothalamic paraventricular and suprachiasmatic nuclei of male and female rats. J Biol Rhythms 30:417–436. 10.1177/0748730415598608 26271538 PMC7697268

[B11] Coria-Lucero CD, Golini RS, Ponce IT, Deyurka N, Anzulovich AC, Delgado SM, Navigatore-Fonzo LS (2016) Rhythmic Bdnf and TrkB expression patterns in the prefrontal cortex are lost in aged rats. Brain Res 1653:51–58. 10.1016/j.brainres.2016.10.019 27771283

[B12] Craske MG, Kircanski K, Zelikowsky M, Mystkowski J, Chowdhury N, Baker A (2008) Optimizing inhibitory learning during exposure therapy. Behav Res Ther 46:5–27. 10.1016/j.brat.2007.10.003 18005936

[B13] Craske MG, Treanor M, Conway CC, Zbozinek T, Vervliet B (2014) Maximizing exposure therapy: an inhibitory learning approach. Behav Res Ther 58:10–23. 10.1016/j.brat.2014.04.006 24864005 PMC4114726

[B14] de Quervain DJ-F, Aerni A, Schelling G, Roozendaal B (2009) Glucocorticoids and the regulation of memory in health and disease. Front Neuroendocrinol 30:358–370. 10.1016/j.yfrne.2009.03.002 19341764

[B15] Dickmeis T, Weger BD, Weger M (2013) The circadian clock and glucocorticoids–interactions across many time scales. Mol Cell Endocrinol 380:2–15. 10.1016/j.mce.2013.05.012 23707790

[B16] Do-Monte FH, Quiñones-Laracuente K, Quirk GJ (2015) A temporal shift in the circuits mediating retrieval of fear memory. Nature 519:460–463. 10.1038/nature14030 25600268 PMC4376623

[B17] Eckel-Mahan KL, Phan T, Han S, Wang H, Chan GCK, Scheiner ZS, Storm DR (2008) Circadian oscillation of hippocampal MAPK activity and cAmp: implications for memory persistence. Nat Neurosci 11:1074–1082. 19160506 10.1038/nn.2174 PMC2772165

[B18] Garfinkel SN, Abelson JL, King AP, Sripada RK, Wang X, Gaines LM, Liberzon I (2014) Impaired contextual modulation of memories in PTSD: an fMRI and psychophysiological study of extinction retention and fear renewal. J Neurosci 34:13435–13443. 10.1523/JNEUROSCI.4287-13.2014 25274821 PMC4262698

[B19] Girgenti MJ, Ghosal S, LoPresto D, Taylor JR, Duman RS (2017) Ketamine accelerates fear extinction via mTORC1 signaling. Neurobiol Dis 100:1–8. 10.1016/j.nbd.2016.12.026 28043916 PMC5907920

[B20] Girotti M, Weinberg MS, Spencer RL (2009) Diurnal expression of functional and clock-related genes throughout the rat HPA axis: system-wide shifts in response to a restricted feeding schedule. Am J Physiol Endocrinol Metab 296:E888–E897. 10.1152/ajpendo.90946.2008 19190255 PMC2670633

[B21] Giustino TF, Maren S (2015) The role of the medial prefrontal cortex in the conditioning and extinction of fear. Front Behav Neurosci 9:298. 10.3389/fnbeh.2015.00298 26617500 PMC4637424

[B22] Hopkins ME, Bucci DJ (2010) Interpreting the effects of exercise on fear conditioning: the influence of time of day. Behav Neurosci 124:868–872. 10.1037/a0021200 21038936 PMC4476371

[B23] Hugues S, Deschaux O, Garcia R (2004) Postextinction infusion of a mitogen-activated protein kinase inhibitor into the medial prefrontal cortex impairs memory of the extinction of conditioned fear. Learn Mem 11:540–543. 10.1101/lm.77704 15466305

[B24] Hui GK, Figueroa IR, Poytress BS, Roozendaal B, McGaugh JL, Weinberger NM (2004) Memory enhancement of classical fear conditioning by post-training injections of corticosterone in rats. Neurobiol Learn Mem 81:67–74. 14670360 10.1016/j.nlm.2003.09.002

[B25] Jacobson L, Akana SF, Cascio CS, Shinsako J, Dallman MF (1988) Circadian variations in plasma corticosterone permit normal termination of adrenocorticotropin responses to stress. Endocrinology 122:1343–1348. 10.1210/endo-122-4-1343 2831028

[B26] Karatsoreos IN, Bhagat S, Bloss EB, Morrison JH, McEwen BS (2011) Disruption of circadian clocks has ramifications for metabolism, brain, and behavior. Proc Natl Acad Sci USA 108:1657–1662. 10.1073/pnas.1018375108 21220317 PMC3029753

[B27] Knapska E, Maren S (2009) Reciprocal patterns of c-Fos expression in the medial prefrontal cortex and amygdala after extinction and renewal of conditioned fear. Learn Mem 16:486–493. 10.1101/lm.1463909 19633138 PMC2726014

[B28] Lai KO, Liang Z, Fei E, Huang H, Ip NY (2015) Cyclin-dependent kinase 5 (Cdk5)-dependent phosphorylation of p70 ribosomal S6 kinase 1 (S6K) is required for dendritic spine morphogenesis. J Biol Chem 290:14637–14646. 10.1074/jbc.M114.627117 25903132 PMC4505530

[B29] Landgraf D, McCarthy MJ, Welsh DK (2014) Circadian clock and stress interactions in the molecular biology of psychiatric disorders. Curr Psychiatry Rep 16:483. 10.1007/s11920-014-0483-7 25135782

[B30] Lin CH, Yeh SH, Lu HY, Gean PW (2003) The similarities and diversities of signal pathways leading to consolidation of conditioning and consolidation of extinction of fear memory. J Neurosci 23:8310–8317. 12967993 10.1523/JNEUROSCI.23-23-08310.2003 PMC6740702

[B31] Liston C, Cichon JM, Jeanneteau F, Jia Z, Chao MV, Gan W-B (2013) Circadian glucocorticoid oscillations promote learning-dependent synapse formation and maintenance. Nat Neurosci 16:698–705. 10.1038/nn.3387 23624512 PMC3896394

[B32] Malek ZS, Sage D, Pévet P, Raison S (2007) Daily rhythm of tryptophan hydroxylase-2 messenger ribonucleic acid within raphe neurons is induced by corticoid daily surge and modulated by enhanced locomotor activity. Endocrinology 148:5165–5172. 10.1210/en.2007-0526 17595225

[B33] Marek R, Xu L, Sullivan RKP, Sah P (2018) Excitatory connections between the prelimbic and infralimbic medial prefrontal cortex show a role for the prelimbic cortex in fear extinction. Nat Neurosci 21:654–658. 10.1038/s41593-018-0137-x 29686260

[B34] Marek T, Fafrowicz M, Golonka K, Mojsa-Kaja J, Oginska H, Tucholska K, Urbanik A, Beldzik E, Domagalik A (2010) Diurnal patterns of activity of the orienting and executive attention neuronal networks in subjects performing a Stroop-like task: a functional magnetic resonance imaging study. Chronobiol Int 27:945–958. 10.3109/07420528.2010.489400 20636208

[B35] Matchock RL, Mordkoff JT (2009) Chronotype and time-of-day influences on the alerting, orienting, and executive components of attention. Exp Brain Res 192:189–198. 10.1007/s00221-008-1567-6 18810396

[B36] Melo I, Ehrlich I (2016) Sleep supports cued fear extinction memory consolidation independent of circadian phase. Neurobiol Learn Mem 132:9–17. 10.1016/j.nlm.2016.04.007 27109918

[B37] Mendoza J, Challet E (2009) Brain clocks: from the suprachiasmatic nuclei to a cerebral network. Neuroscientist 15:477–488. 10.1177/1073858408327808 19224887

[B38] Meuret AE, Rosenfield D, Bhaskara L, Auchus R, Liberzon I, Ritz T, Abelson JL (2016) Timing matters: endogenous cortisol mediates benefits from early-day psychotherapy. Psychoneuroendocrinology 74:197–202. 10.1016/j.psyneuen.2016.09.008 27664810

[B39] Milad MR, Rosenbaum BL, Simon NM (2014) Neuroscience of fear extinction: implications for assessment and treatment of fear-based and anxiety related disorders. Behav Res Ther 62:17–23. 10.1016/j.brat.2014.08.006 25204715

[B40] Morris MC, Compas BE, Garber J (2012) Relations among posttraumatic stress disorder, comorbid major depression, and HPA function: a systematic review and meta-analysis. Clin Psychol Rev 32:301–315. 10.1016/j.cpr.2012.02.002 22459791 PMC3340453

[B41] Mukherjee S, Coque L, Cao J-L, Kumar J, Chakravarty S, Asaithamby A, Graham A, Gordon E, Enwright JF, DiLeone RJ, Birnbaum SG, Cooper DC, McClung CA (2010) Knockdown of Clock in the ventral tegmental area through RNA interference results in a mixed state of mania and depression-like behavior. Biol Psychiatry 68:503–511. 10.1016/j.biopsych.2010.04.031 20591414 PMC2929276

[B42] Osterlund CD, Jarvis E, Chadayammuri A, Unnithan R, Weiser MJ, Spencer RL (2011) Tonic, but not phasic corticosterone, constrains stress activated extracellular-regulated-kinase 1/2 immunoreactivity within the hypothalamic paraventricular nucleus. J Neuroendocrinol 23:1241–1251. 10.1111/j.1365-2826.2011.02220.x 21929693 PMC3220802

[B43] Pace-Schott EF, Spencer RMC, Vijayakumar S, Ahmed NAK, Verga PW, Orr SP, Pitman RK, Milad MR (2013) Extinction of conditioned fear is better learned and recalled in the morning than in the evening. J Psychiatr Res 47:1776–1784. 10.1016/j.jpsychires.2013.07.027 23992769 PMC3791331

[B44] Paxinos G, Watson C (1986) The rat brain in stereotaxic coordinates, Ed 2 San Diego, CA: Academic Press.

[B45] Pellman BA, Kim E, Reilly M, Kashima J, Motch O, de la Iglesia HO, Kim JJ (2015) Time-specific fear acts as a non-photic entraining stimulus of circadian rhythms in rats. Sci Rep 5:14916. 10.1038/srep14916 26468624 PMC4606733

[B46] Perez-Cruz C, Simon M, Flügge G, Fuchs E, Czéh B (2009) Diurnal rhythm and stress regulate dendritic architecture and spine density of pyramidal neurons in the rat infralimbic cortex. Behav Brain Res 205:406–413. 10.1016/j.bbr.2009.07.021 19643147

[B47] Pugh CR, Tremblay D, Fleshner M, Rudy JW (1997) A selective role for corticosterone in contextual-fear conditioning. Behav Neurosci 111:503–511. 9189265

[B48] Roozendaal B (2002) Stress and memory: opposing effects of glucocorticoids on memory consolidation and memory retrieval. Neurobiol Learn Mem 78:578–595. 12559837 10.1006/nlme.2002.4080

[B49] Rudy JW, Pugh CR (1998) Time of conditioning selectively influences contextual fear conditioning: further support for a multiple-memory systems view of fear conditioning. J Exp Psychol Anim Behav Process 24:316–324. 9679307 10.1037//0097-7403.24.3.316

[B50] Schottenbauer MA, Glass CR, Arnkoff DB, Tendick V, Gray SH (2008) Nonresponse and dropout rates in outcome studies on PTSD: review and methodological considerations. Psychiatry 71:134–168. 10.1521/psyc.2008.71.2.134 18573035

[B51] Serita T, Fukushima H, Kida S (2017) Constitutive activation of CREB in mice enhances temporal association learning and increases hippocampal CA1 neuronal spine density and complexity. Sci Rep 7:42528. 10.1038/srep42528 28195219 PMC5307365

[B52] Snider KH, Dziema H, Aten S, Loeser J, Norona FE, Hoyt K, Obrietan K (2016) Modulation of learning and memory by the targeted deletion of the circadian clock gene Bmal1 in forebrain circuits. Behav Brain Res 308:222–235. 10.1016/j.bbr.2016.04.027 27091299 PMC5344043

[B53] Snider KH, Sullivan KA, Obrietan K (2018) Circadian regulation of hippocampal-dependent memory: circuits, synapses, and molecular mechanisms. Neural Plast 2018:7292540. 10.1155/2018/7292540 29593785 PMC5822921

[B54] Soshi T, Kuriyama K, Aritake S, Enomoto M, Hida A, Tamura M, Kim Y, Mishima K (2010) Sleep deprivation influences diurnal variation of human time perception with prefrontal activity change: a functional near-infrared spectroscopy study. PLoS One 5:e8395. 10.1371/journal.pone.0008395 20049334 PMC2797606

[B55] Sotres-Bayon F, Cain CK, Ledoux JE (2006) Brain mechanisms of fear extinction: historical perspectives on the contribution of prefrontal cortex. Biol Psychiatry 60:329–336. 10.1016/j.biopsych.2005.10.012 16412988

[B56] Sotres-Bayon F, Quirk GJ (2010) Prefrontal control of fear: more than just extinction. Curr Opin Neurobiol 20:231–235. 10.1016/j.conb.2010.02.005 20303254 PMC2878722

[B57] Spencer RL, Deak T (2017) A users guide to HPA axis research. Physiol Behav 178:43–65. 10.1016/j.physbeh.2016.11.014 27871862 PMC5451309

[B58] Spencer RL, Chun LE, Hartsock MJ, Woodruff ER (2018) Glucocorticoid hormones are both a major circadian signal and major stress signal: how this shared signal contributes to a dynamic relationship between the circadian and stress systems. Front Neuroendocrinol 49:52–71. 10.1016/j.yfrne.2017.12.005 29288075

[B59] Spencer S, Falcon E, Kumar J, Krishnan V, Mukherjee S, Birnbaum SG, McClung CA (2013) Circadian genes Period 1 and Period 2 in the nucleus accumbens regulate anxiety-related behavior. Eur J Neurosci 37:242–250. 10.1111/ejn.12010 23039899 PMC3711746

[B60] Sylvester CM, Krout KE, Loewy AD (2002) Suprachiasmatic nucleus projection to the medial prefrontal cortex: a viral transneuronal tracing study. Neuroscience 114:1071–1080. 12379260 10.1016/s0306-4522(02)00361-5

[B61] Tuscher JJ, Luine V, Frankfurt M, Frick KM (2016) Estradiol-mediated spine changes in the dorsal hippocampus and medial prefrontal cortex of ovariectomized female mice depend on ERK and mTOR activation in the dorsal hippocampus. J Neurosci 36:1483–1489. 10.1523/JNEUROSCI.3135-15.2016 26843632 PMC4737764

[B62] van Minnen A, Arntz A, Keijsers GPJ (2002) Prolonged exposure in patients with chronic PTSD: predictors of treatment outcome and dropout. Behav Res Ther 40:439–457. 10.1016/S0005-7967(01)00024-9 12002900

[B63] Vanelzakker MB, Kathryn Dahlgren M, Caroline Davis F, Dubois S, Shin LM (2014) From Pavlov to PTSD: the extinction of conditioned fear in rodents, humans, and anxiety disorders. Neurobiol Learn Mem 113:3–18. 10.1016/j.nlm.2013.11.014 24321650 PMC4156287

[B64] Watts AG, Swanson LW, Sanchez-Watts G (1987) Efferent projections of the suprachiasmatic nucleus: I. Studies using anterograde transport of *Phaseolus vulgaris* leucoagglutinin in the rat. J Comp Neur 258:204–229. 10.1002/cne.902580204 3294923

[B65] Woodruff ER, Chun LE, Hinds LR, Spencer RL (2016) Diurnal corticosterone presence and phase modulate clock gene expression in the male rat prefrontal cortex. Endocrinology 157:1522–1534. 10.1210/en.2015-1884 26901093 PMC4816727

[B66] Woodruff ER, Greenwood BN, Chun LE, Fardi S, Hinds LR, Spencer RL (2015) Adrenal-dependent diurnal modulation of conditioned fear extinction learning. Behav Brain Res 286:249–255. 10.1016/j.bbr.2015.03.006 25746455 PMC4465528

[B67] Xiong H, Cassé F, Zhou Y, Zhou M, Xiong Z-Q, Joëls M, Martin S, Krugers HJ (2015) mTOR is essential for corticosteroid effects on hippocampal AMPA receptor function and fear memory. Learn Mem 22:577–583. 10.1101/lm.039420.115 26572647 PMC4749735

[B68] Yehuda R, Bierer LM, Pratchett LC, Lehrner A, Koch EC, Van Manen JA, Flory JD, Makotkine I, Hildebrandt T (2015) Cortisol augmentation of a psychological treatment for warfighters with posttraumatic stress disorder: randomized trial showing improved treatment retention and outcome. Psychoneuroendocrinology 51:589–597. 10.1016/j.psyneuen.2014.08.004 25212409

[B69] Zuj DV, Palmer MA, Hsu C-MK, Nicholson EL, Cushing PJ, Gray KE, Felmingham KL (2016) Impaired fear extinction associated with PTSD increases with hours-since-waking. Depress Anxiety 33:203–210. 10.1002/da.22463 26744059

